# Supplementary material for: MreB polymers and curvature localization are enhanced by RodZ and predict E. coli's cylindrical uniformity
Source: Nat Commun. 2018 Jul 18;9:2797. doi: 10.1038/s41467-018-05186-5 (PMC6052060; doi:10.1038/s41467-018-05186-5)
Supplement: Supplementary file 1 — Supplementary Information [file 41467_2018_5186_MOESM1_ESM.pdf]

MreB polymers and curvature localization are enhanced by RodZ and predict *E. coli’s* cylindrical uniformity

Bratton, Benjamin P. et al

Supplementary Information

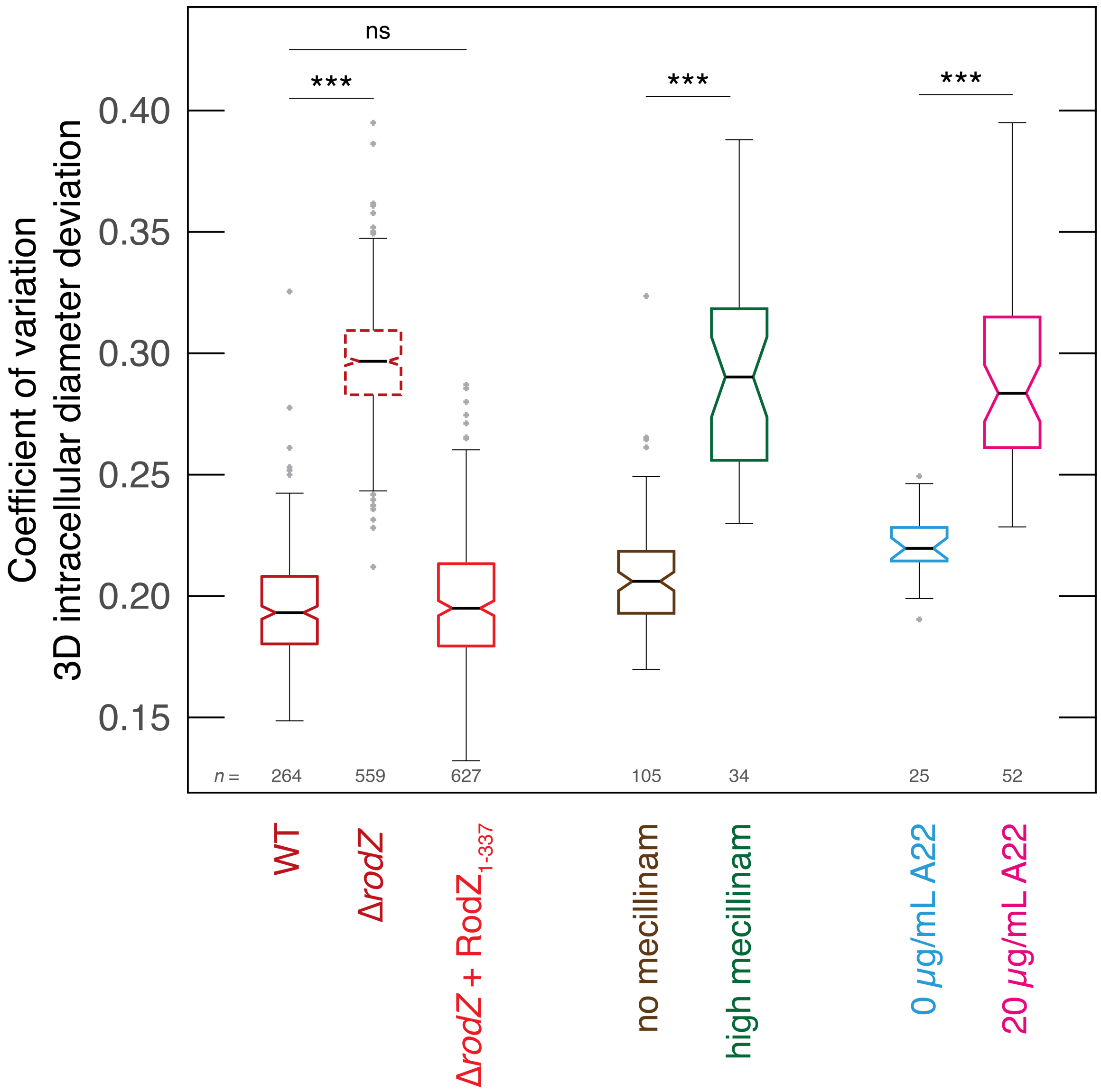

Supplementary Fig. 1. Cylindrical uniformity is disrupted by deletion of *rodZ*, or in the presence of mecillinam or A22. Box and whisker plots show the median (bold black line), 95% CI of the median (notch), quartiles Q1 and Q3 (colored lines), range (whiskers), and outliers more than one IQR outside of Q1 or Q3 (gray dots) for cell shape perturbations. *P*-values from Student's *t*-test comparisons, ns $P > 0.05$, *** $P \leq 0.001$. These experiments were performed on three separate days and the data were pooled.

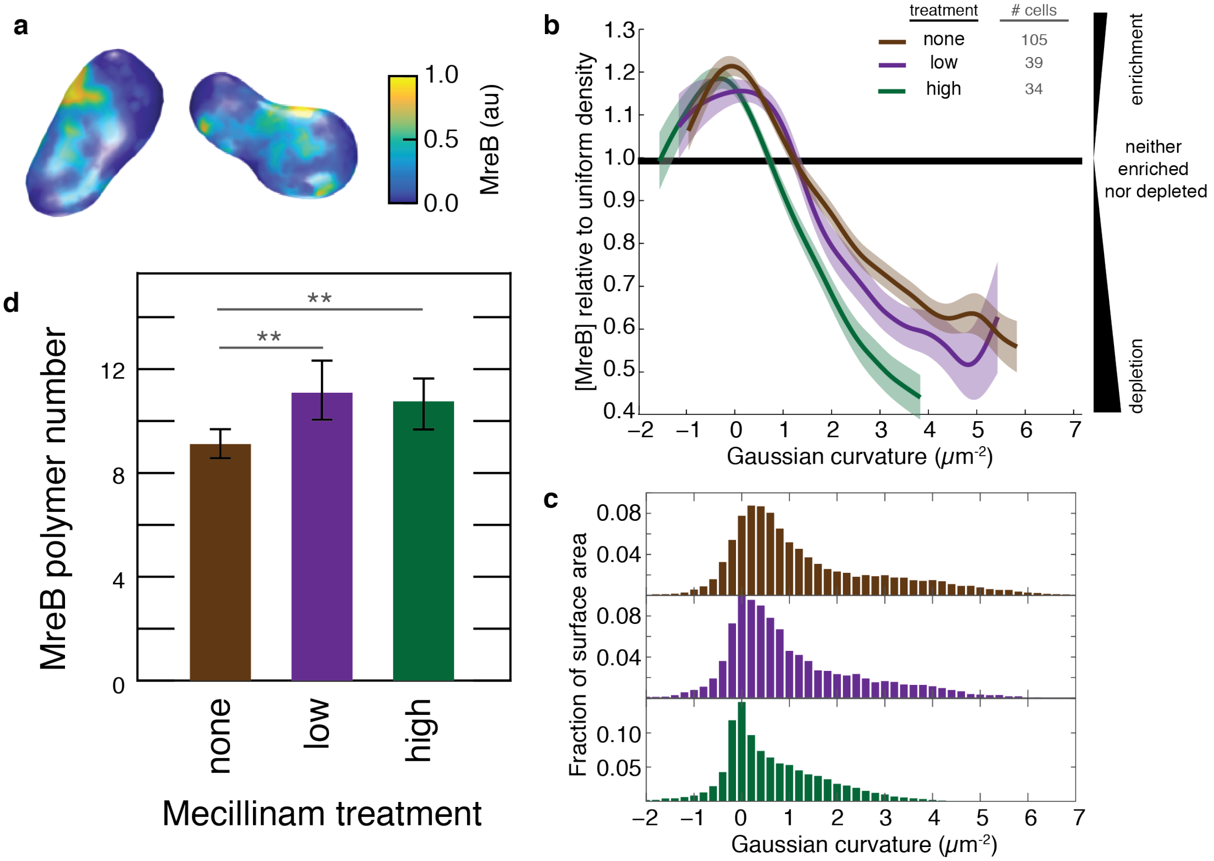

Supplementary Fig. 2. Mecillinam treated cells have geometrically-localized MreB. Cells were either treated with no mecillinam, low levels (0.025-0.050 μg/ml), or high levels (0.1-0.15 μg/ml) for 3-4 hours. Cells were imaged on pads absent of any drug in all cases. **a** 3D reconstructions of cells treated with high levels of mecillinam displaying MreB localization. **b** Curvature enrichment plot of mecillinam treated cells. The curve for each condition is a cubic smoothing spline and is truncated using a probability threshold for extreme curvatures of $p > 5\times10^{-3}$. Because the shape of each strain is different, the ranges of curvatures plotted for each strain are different. Mecillinam does not have an effect on curvature localization. Shaded area indicates ± 1 standard error of the mean. **c** Histograms of the distribution of curvatures found in mecillinam treated cells. **d** Mecillinam rounded cells do not display a decreased number of polymers. Error bars represent 95% confidence intervals from bootstrap analysis. **b-d** These experiments were performed on three separate days and the data were pooled. *P*-values from Student's *t*-test comparisons, ** $P \leq 0.01$.

a

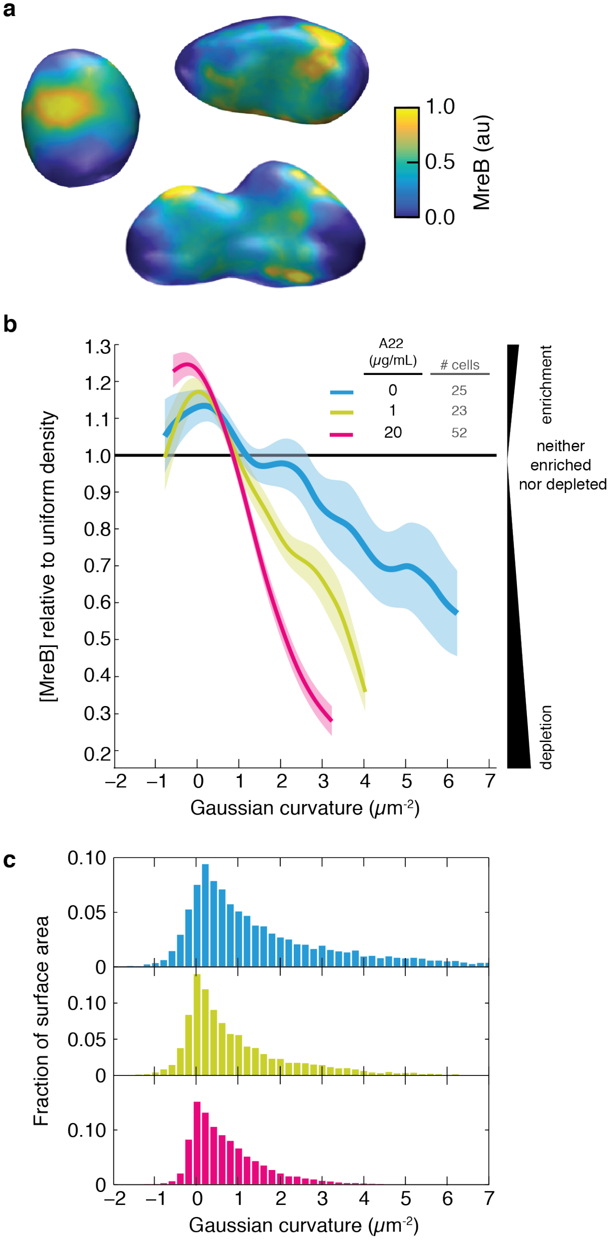

Supplementary Fig. 3. A22 treated cells have geometrically-localized MreB. Cells were either treated with methanol only, low levels (1 µg/ml), or high levels (20 µg/ml) for one hour and then gently washed into fresh LB. Cells were imaged on pads absent of any A22 in all cases. **a** 3D reconstructions of cells treated with high levels of A22 displaying MreB localization. **b** Curvature enrichment plot of A22 treated cells. The curve for each condition is a cubic smoothing spline and is truncated using a probability threshold for extreme curvatures of $p > 5x10^{-3}$. Because the shape of each strain is different, the ranges of curvatures plotted for each strain are different. Mecillinam does not have an effect on curvature localization. A22 treatment leads to a more pronounced geometric enrichment of MreB. Shaded area indicates ± 1 standard error of the mean. **c** Histograms of the distribution of curvatures found in A22 treated cells. These experiments were performed on three separate days and the data were pooled.

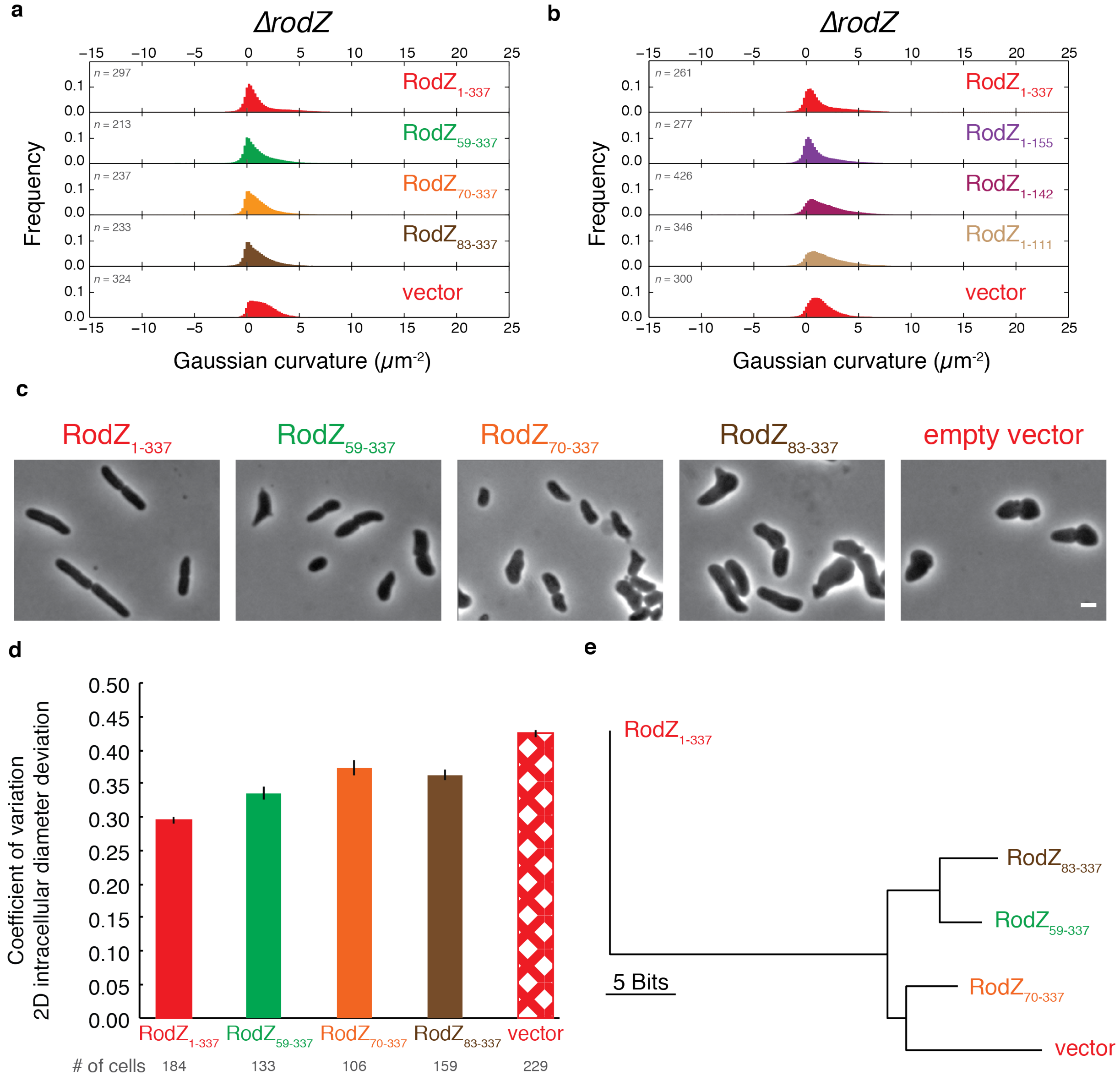

Supplementary Fig. 4. Cell shape analysis of RodZ cytoplasmic truncations and representative curvatures found in RodZ truncations. **a** Histogram of the distribution of curvatures found in RodZ cytoplasmic truncations. **b** Distribution of curvatures found in RodZ periplasmic truncations. **c** 2D images of RodZ cytoplasmic truncations. Scale bar is 2 μm. **d** Intracellular diameter deviation of RodZ cytoplasmic truncations. Error bars represent 95% confidence intervals from bootstrap analysis. **e** Tree showing the differences between strains that express full length or cytoplasmic RodZ truncations by SPACECRAFT analysis. These experiments were performed on three separate days and the data were pooled.

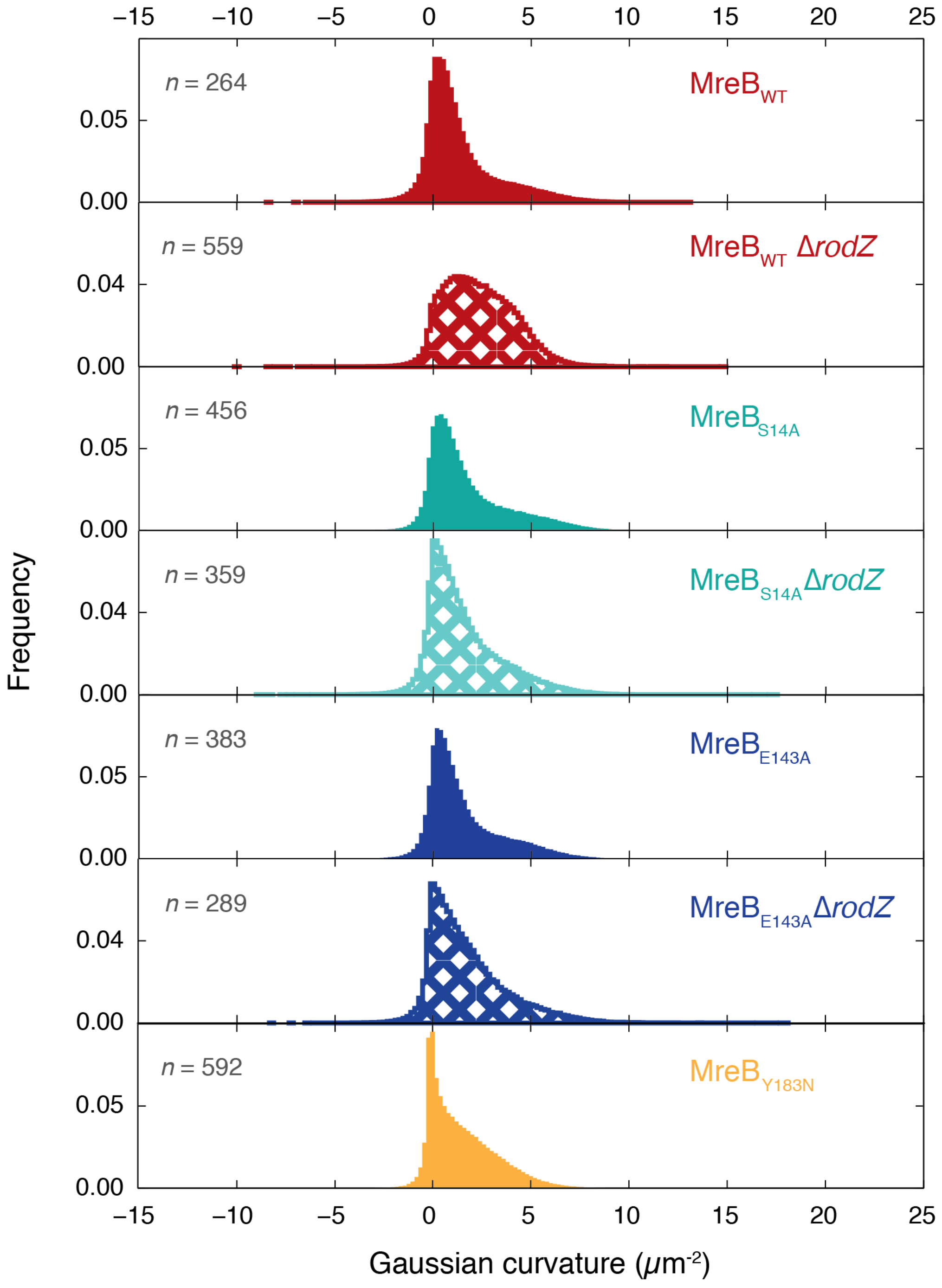

Supplementary Fig. 5. Distribution of curvatures found in MreB point mutants. Histograms of the distribution of curvatures found in MreB point mutants in the presence and absence of RodZ. Number of cells sampled for each condition is listed in gray in each sub-panel. These experiments were performed on three separate days and the data were pooled.

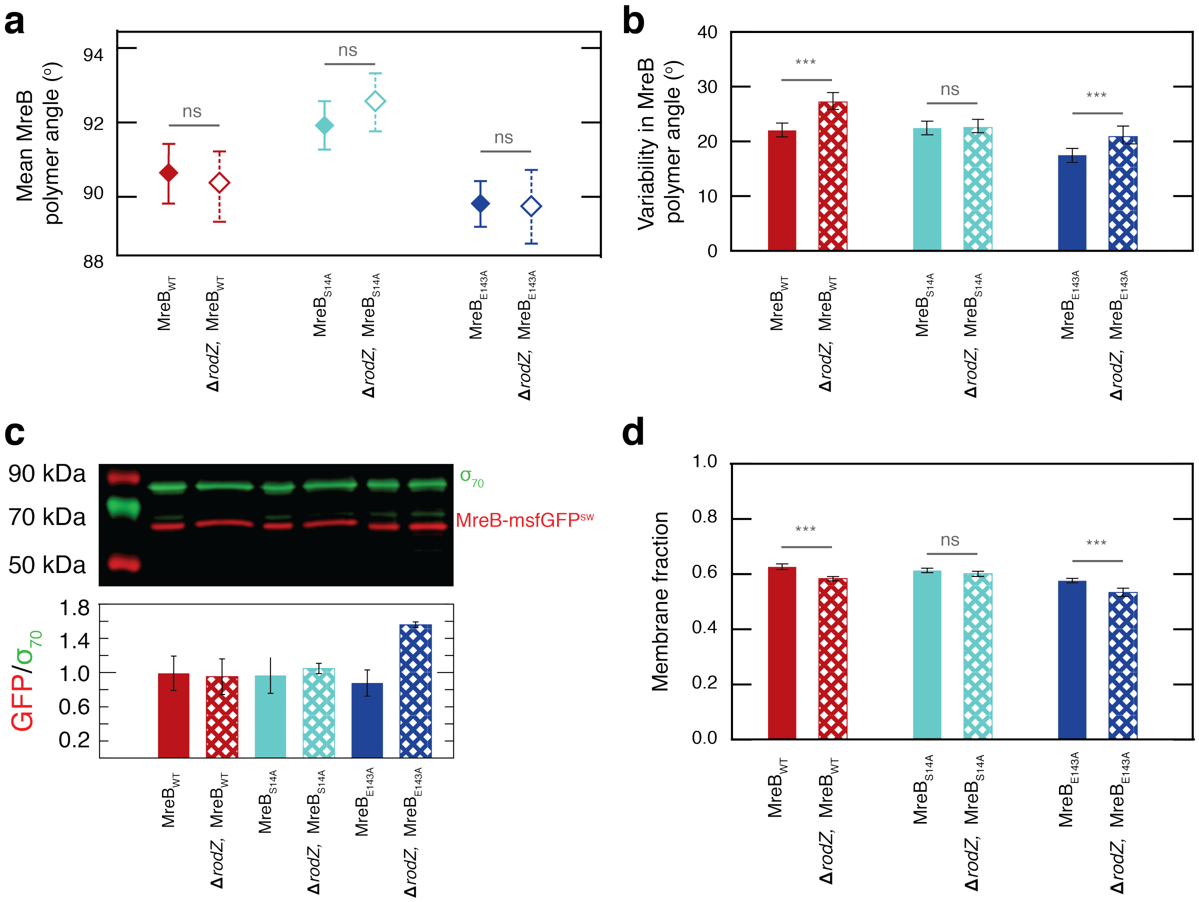

Supplementary Fig. 6. RodZ modulates MreB parameters in an allele specific manner. **a** Average MreB angle is not affected by RodZ, but is affected by MreB allele. Error bars represent 95% confidence intervals from bootstrap analysis. **b** The variation in MreB angle, as measured by inter-quartile range, is larger in the absence of RodZ in WT cells and $MreB_{E143A}$ cells but not $MreB_{S14A}$. Error bars represent 95% confidence intervals from bootstrap analysis. **c** Western blot indicating that steady-state MreB levels are not affected by RodZ, except in $MreB_{E143A}$ cells. Error bars represent ± SEM. **d** The percent of MreB on the membrane is decreased in the absence of RodZ for both WT and $MreB_{E143A}$ but not $MreB_{S14A}$. Error bars represent 95% confidence intervals from bootstrap analysis. *P*-values from Student's *t*-test comparisons, ns $P > 0.05$, *** $P \leq 0.001$. **a**, **b**, **d** These experiments were performed on three separate days and the data were pooled. **a**, **b** Number of polymers in each pooled dataset is displayed above each bar. **d** Number of cells in each pooled dataset is displayed above each bar.

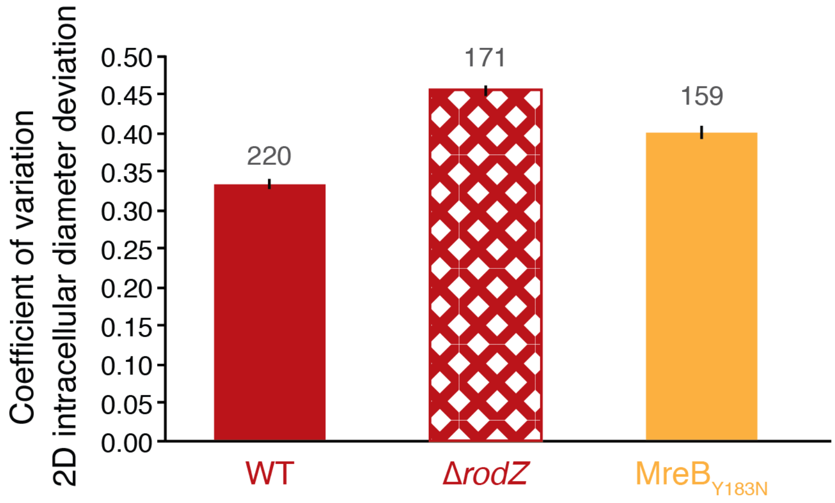

Supplementary Fig. 7. MreB$_{Y183N}$ is not rod shaped. Intracellular diameter deviation of MreB$_{Y183N}$. Error bars represent 95% confidence intervals from bootstrap analysis. These experiments were performed on three separate days and the data were pooled and the number of cells in each pooled dataset is displayed above each bar.

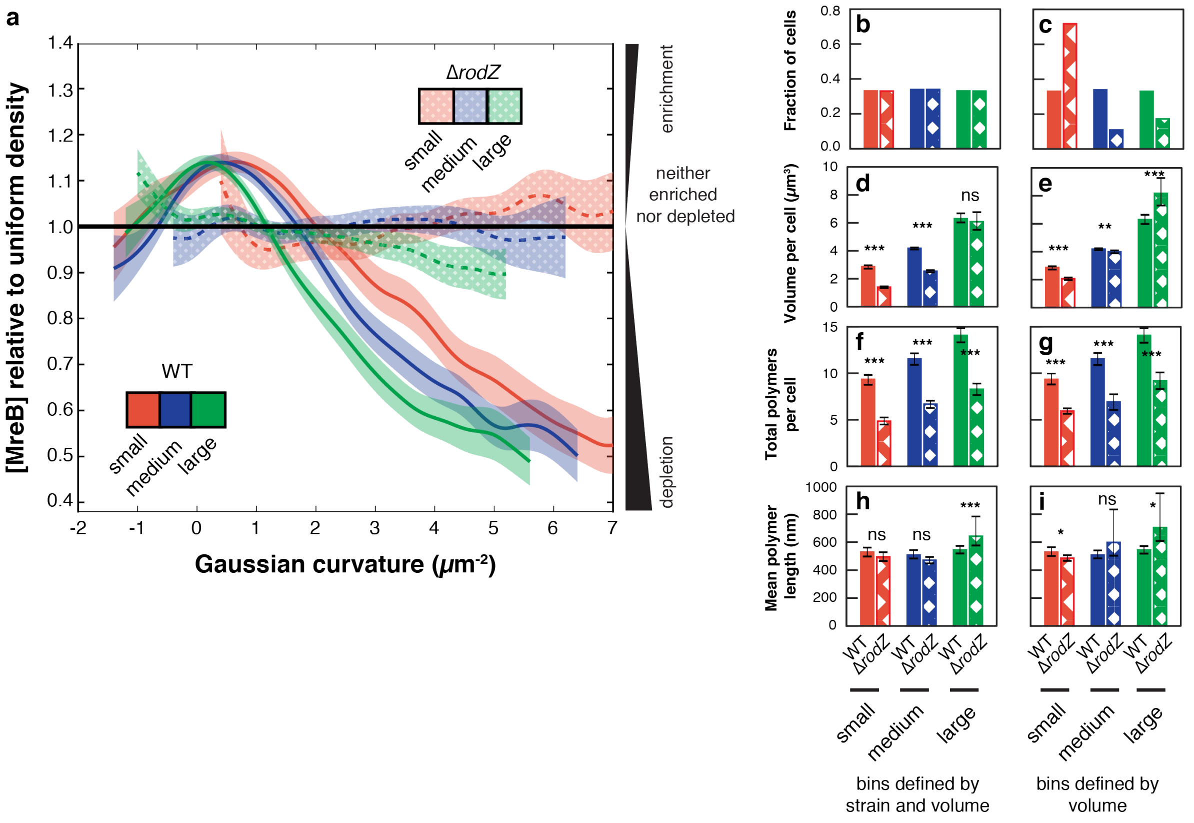

Supplementary Fig. 8. RodZ has a greater effect than volume on MreB polymer number. **a** MreB curvature enrichment plot for WT and Δ*rodZ* cells binned by size. The curve for each condition is a cubic smoothing spline and is truncated using a probability threshold for extreme curvatures of $p > 5\text{x}10^{-3}$. Because the shape of each strain is different, the ranges of curvatures plotted for each strain are different. Mecillinam does not have an effect on curvature localization. WT cells are represented by solid bars and Δ*rodZ* cells by dashed lines. Shaded area indicates ± 1 standard error of the mean. **b**-**c** The fraction of cells in each bin for each strain. **d**-**e** The volume/cell of each strain. **f**-**g** The number of polymers/cell for each strain in each bin. **h**-**i** The mean polymer length for each bin. **a**, **b**, **d**, **f**, **h** Each strain was separated into 3 equal bins based on the volume distribution of that strain. **c**, **e**, **g**, **i** WT and Δ*rodZ* cells were separated into bins based on WT cell volume. **b**-**i** Error bars represent 95% confidence intervals from bootstrap analysis. *P*-values from Student's *t*-test comparisons, ns $P > 0.05$, * $P \leq 0.05$, ** $P \leq 0.01$, *** $P \leq 0.001$. These experiments were performed on three separate days and the data were pooled.

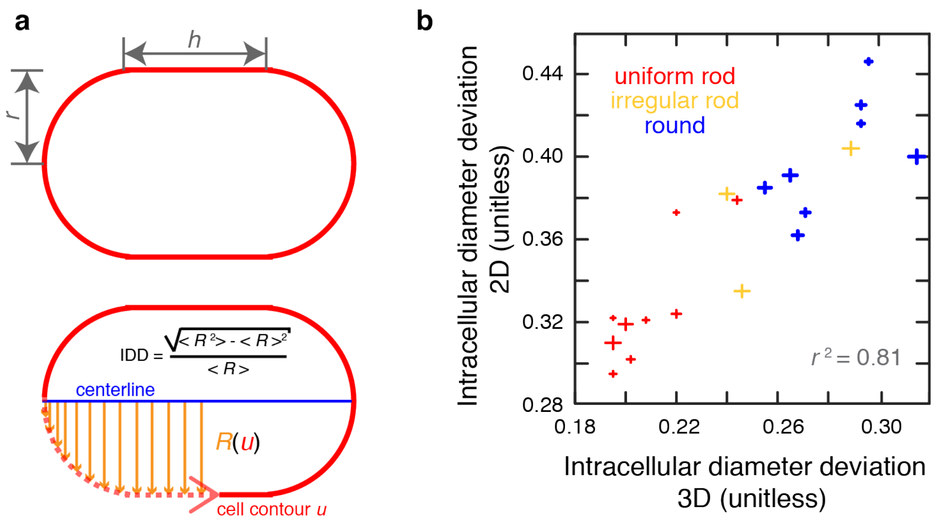

Supplementary Fig. 9. Intracellular diameter deviation is a quantitative measurement of cylindrical uniformity. **a** IDD is calculated as coefficient of variation of the distance from the centerline to the cell surface and is weighted by the length along the contour in 2D and by surface area in 3D. **b** 2D intracellular diameter deviation and 3D intracellular diameter deviation measurements on different sample preparations of the same strain are highly correlated with each other. Error bars are the bootstrap 95% confidence intervals of the mean. These experiments were performed on three separate days and the data were pooled.

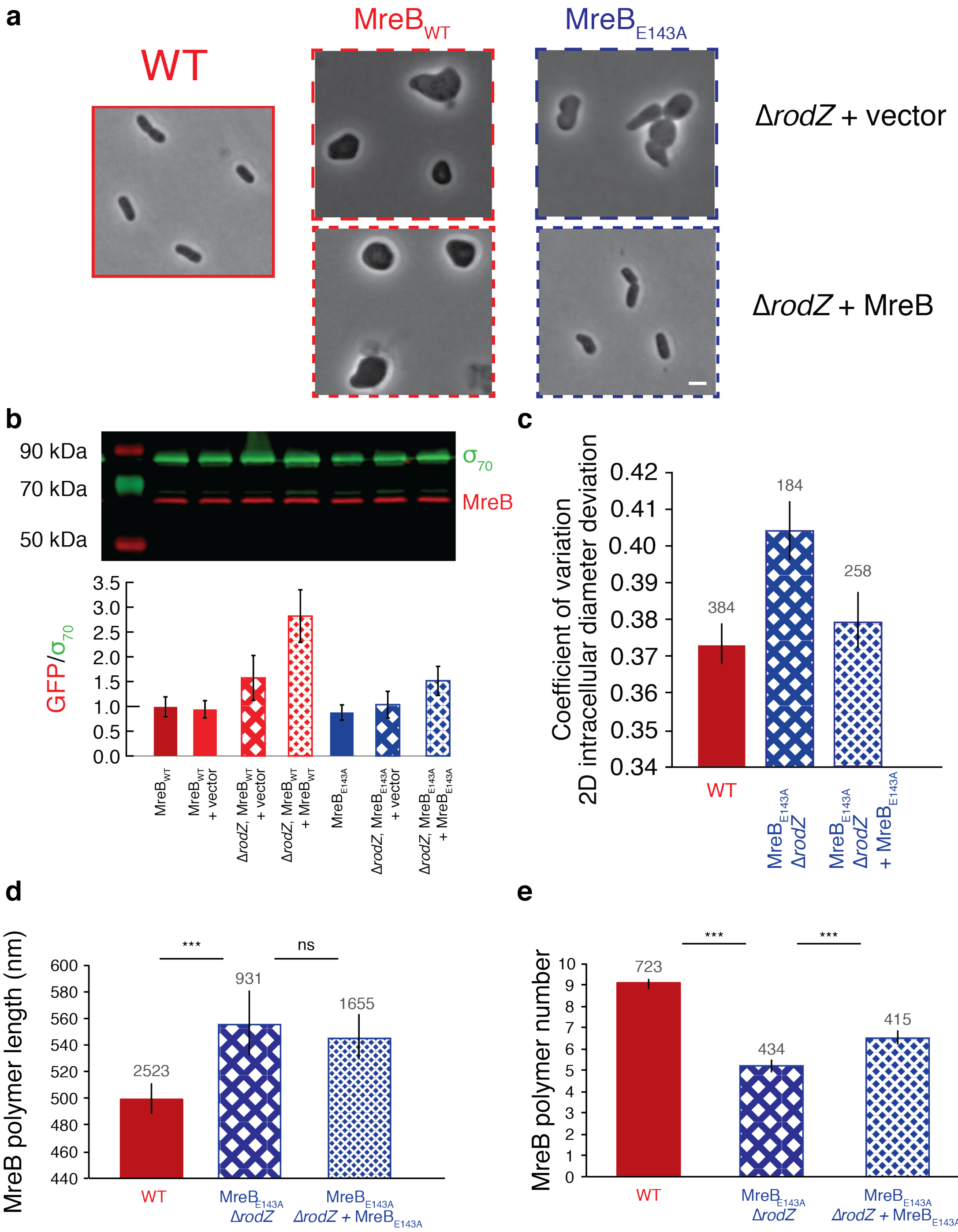

Supplementary Fig. 10. Ectopic expression of MreB$_{E143A}$ restores cell shape in MreB$_{E143A}$Δ*rodZ* but ectopic expression of MreB$_{WT}$ does not restore cell shape in MreB$_{WT}$Δ*rodZ.* **a** 2D images of WT, MreB$_{WT}$Δ*rodZ* with and without ectopic expression of MreB$_{WT}$, and MreB$_{E143A}$Δ*rodZ* with and without ectopic expression of MreB$_{E143A}$. Scale bar is 2 µm. **b** Western blot indicating the relative levels of MreB levels following ectopic expression. The bars are the mean ± 1 standard deviation across 3-10 biological replicates after removing outliers from each distribution (|*z*-score|>1.5). **c** Intracellular diameter deviation of WT cells and MreB$_{E143A}$Δ*rodZ* cells with and without

ectopic expression of $MreB_{E143A}$. The number of cells in each pooled dataset is displayed above each bar. **d** MreB polymer length measurements of WT cells and $MreB_{E143A}$Δ*rodZ* cells with and without ectopic expression of $MreB_{E143A}$. The number of polymers in each pooled dataset is displayed above each bar. **e** The number of polymers in WT cells $MreB_{E143A}$Δ*rodZ* cells with and without ectopic expression of $MreB_{E143A}$. The number of cells in each pooled dataset is displayed above each bar. **c**-**e** Error bars are 95% confidence intervals from bootstrap analysis. *P*-values from Student's *t*-test comparisons, ns P > 0.05, *** $P \leq 0.001$. These experiments were performed on three separate days and the data were pooled.

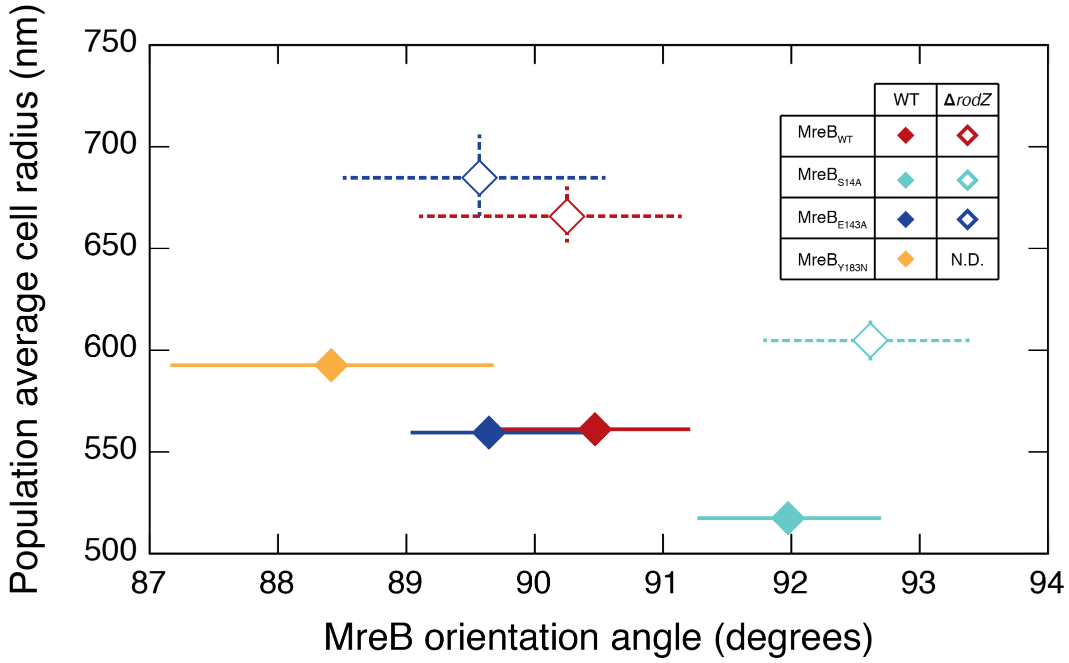

Supplementary Fig. 11. There is a negative correlation between MreB angle and cell width. Deleting *rodZ* increase the average cell radius but does not remove the negative correlation. Error bars represent 95% confidence intervals from bootstrap analysis. These experiments were performed on three separate days and the data were pooled.

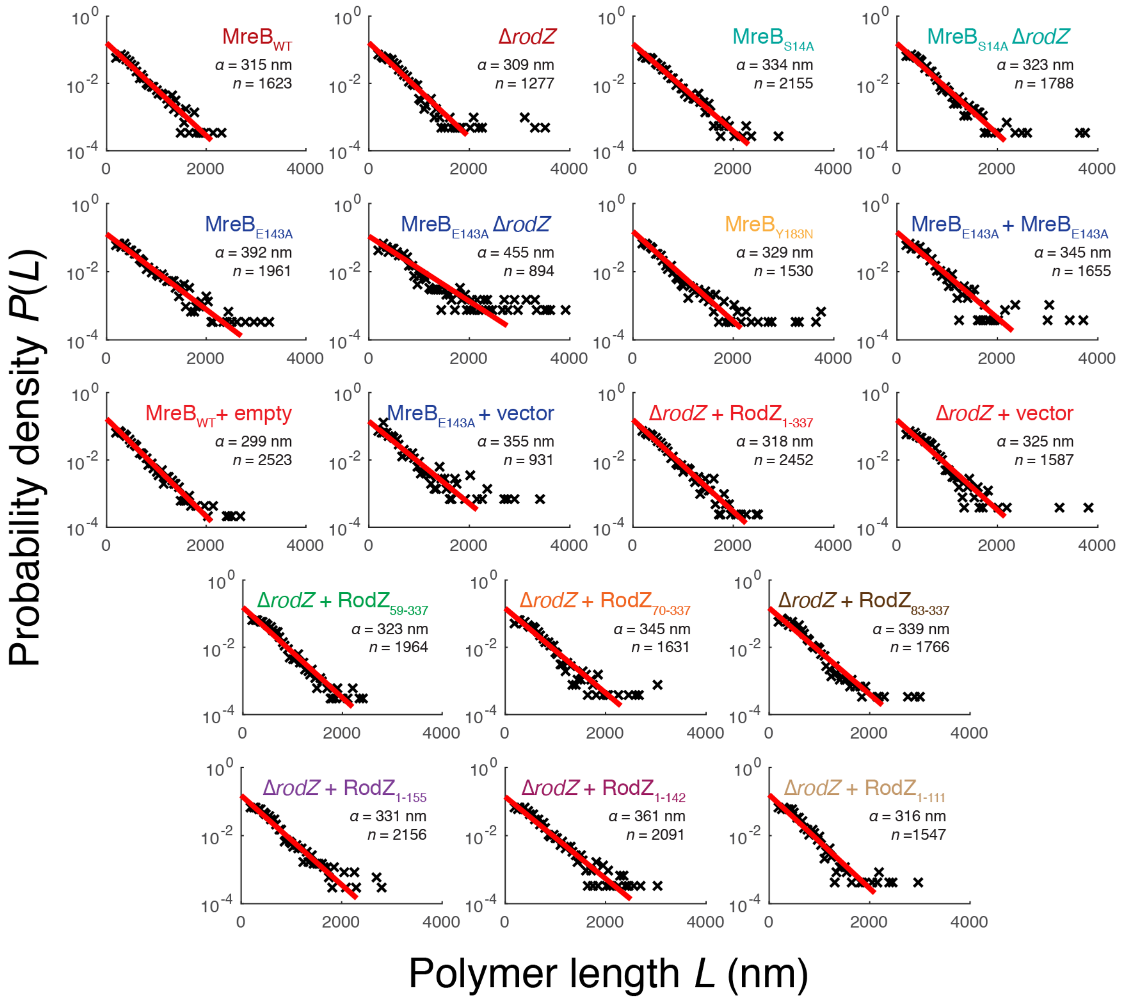

Supplementary Fig. 12. Distribution of MreB polymer lengths follow a thresholded exponential. Black crosses represent the probability distribution of measured polymer lengths greater than 200 nm. The distributions are normalized by the total number of polymers, although those less than 200 nm are not plotted. Red lines are predicted exponential distributions with an exponential decay length exactly 200 nm less than the average polymer length. The name of the strain, the decay length and the number of polymers included in the analysis are listed as insets in each panel. These experiments were performed on three separate days and the data were pooled.

Supplementary Table 1. Strain list.

Strain	Chromosome*	Plasmid†	Ref
ZG231	DH5α	pTrc99a	1
NO56	*ΔrodZ (cam), mreB-msfGFPsw* (kan)	mCherry (tet)	2
NO56 pTrc	*ΔrodZ (cam), mreB-msfGFPsw* (kan)	pTrc99A (amp), mCherry (tet)	This paper
NO83	*mreB-msfGFP*^{sw} (kan)	mCherry (cam)	2
RM481	*ΔrodZ* (*cam*), *mreB-msfGFPsw* (kan)	pTrc99A + CYFP-RodZ1-155 (amp), mCherry (tet)	2
RM482	*ΔrodZ* (*cam*), *mreB-msfGFPsw* (kan)	pTrc99A + CYFP-RodZ1-142 (amp), mCherry (tet)	2
RM483	*ΔrodZ* (*cam*), *mreB-msfGFPsw* (kan)	pTrc99A + CYFP-RodZ1-111 (amp), mCherry (tet)	2
RM474	*ΔrodZ* (*cam*), *mreBE143A-msfGFPSW* (kan)	mCherry (tet)	This paper
E143A	*mreBE143A-msfGFPSW* (kan)	mCherry (tet)	3
RM478	*ΔrodZ* (*cam*), *mreBS14A-msfGFPSW* (kan)	mCherry (tet)	2
RM489	*mreBS14A-msfGFPSW* (kan)	mCherry (cam)	2
RM510	*ΔrodZ* (*cam*), *mreB-msfGFPsw* (kan)	pTrc99A + CYFP-RodZ1-337 (amp), mCherry (tet)	2
RM523	*ΔrodZ* (*cam*)	pTrc99A + CYFP-RodZ83-337 (amp), mCherry (tet)	2
RM529	*ΔrodZ* (*cam*), *mreB*-msfGFPsw	pTrc99A + CYFP-RodZ59-337 (amp), mCherry (tet)	This paper
RM541	*ΔrodZ* (*cam*), *mreB*-msfGFPsw	pTrc99A + CYFP-RodZ70-337 (amp), mCherry (tet)	This paper
RM581	*ΔrodZ*, *mreB*E143A-msfGFPsw	pTrc99A +MreBE143A-msfGFPsw (amp), mCherry (tet)	This paper
RM582	*mreB*-msfGFPsw,	pTrc99A (amp), mCherry (cam)	This paper
RM583	*ΔrodZ*, *mreB*E143A-msfGFPsw	pTrc99A (amp), mCherry (tet)	This paper
Y183N	*mreBY183N-msfGFPSW* (kan)	mCherry (tet)	This paper
RM593	*ΔrodZ (cam), mreB-msfGFPsw* (kan)	pTrc99A +MreB-msfGFPsw (amp), mCherry (tet)	This paper

*Unless otherwise noted, the parental genotype is MG1655 (CGSC 7740).
†Strains used for reconstructing 3D cell shape contain a plasmid constitutively expressing mCherry in the cytoplasm. Depending on the strain background, this plasmid either carries a cassette for tetracycline (tet) or chloramphenicol (cam) resistance.

Supplementary Table 2. List of MreB parameters and LASSO coefficients

MreB parameter	Group*	Non-zero leave-one-out LASSO analyses, λ = 0.02†	Minimal model regression coefficients‡
total polymer length per cell, >200 nm (nm)	A	14/18	0
total polymer length per cell, >200 nm, (nm) / surface area (nm^2)	A	0	0
total polymer length per cell, >200 nm, (nm) / volume (nm^3)	A	13/18	$-91 \times 10^3 \pm 9 \times 10^3$
number of polymers, >200 nm, per cell	B	0	0
number of polymers, >200 nm, per cell / surface area (nm^2)	B	0	0
number of polymers, >200 nm, per cell / volume (nm^3)	B	2/18	0
number of polymers per cell	B	16/18	$-11.3 \times 10^{-3} \pm 0.4 \times 10^{-3}$
number of polymers per cell / surface area (nm^2)	B	0	0
number of polymers per cell / volume (nm^3)	B	0	0
average MreB enrichment, Gaussian curvatures < 2 μm^{-2}	C	0	-0.26 ± 0.01
average MreB enrichment, Gaussian curvatures ≥ 2 μm^{-2}	C	17/18	0
Difference in MreB enrichment, (Gaussian curvatures < 2 μm^{-2}) –(Gaussian curvatures ≥ 2 μm^{-2})	C	14/18	0
mean polymer angle (radians)	D	0	0
Interquartile range of polymer angle (radians)	D	0	0
membrane fraction	D	0	0
mean polymer length, >200 nm (nm)	D	0	0
mean polymer length (nm) >200 nm / surface area (nm^2)	D	0	0
mean polymer length (nm) >200 nm / volume (nm^3)	D	0	0
characteristic length of polymer (nm)	D	0	0
characteristic length of polymer (nm) / surface area (nm^2)	D	0	0
characteristic length of polymer (nm) / volume (nm^3)	D	0	0

*The parameters that were used as inputs to the LASSO regression were divided into four groups based on the types of properties that they represent. Group A, combined length of MreB polymers; group B, number of MreB polymers; group C, MreB curvature localization; group D, all others.

†The LASSO regression was performed omitting a single comparison. Not all iterations of this yielded the same parameters in the minimal model, but no parameters from group D were ever in this minimal model.

‡Of all the possible three-parameter models with no more than one parameter from each of groups ABC (any number of D allowed), this set of coefficients has the lowest mean squared error in 10-fold cross-validation.

Supplementary Table 3. List of *P*-values from a two-tailed unequal variance Student's *t*-test.

polymer length		*P*-value
Strain 1	Strain 2	
WT	Δ*rodZ*	>0.05
WT	MreB$_{S14r}$	>0.05
WT	MreB$_{E143A}$	<.001
Δ*rodZ*	MreB$_{S14A}$ Δ*rodZ*	>0.05
Δ*rodZ*	MreB$_{E143A}$ Δ*rodZ*	<.001
MreB$_{S14A}$	MreB$_{S14A}$ Δ*rodZ*	>0.05
MreB$_{E143A}$	MreB$_{E143A}$ Δ*rodZ*	<0.01

polymer number		*P*-value
Strain 1	Strain 2	
WT	Δ*rodZ*	<0.001
WT	MreB$_{S14A}$	<0.001
WT	MreB$_{E143A}$	<0.001
Δ*rodZ*	MreB$_{S14A}$ Δ*rodZ*	<0.001
Δ*rodZ*	MreB$_{E143A}$ Δ*rodZ*	<0.001
MreB$_{S14A}$	MreB$_{S14A}$ Δ*rodZ*	>0.05
MreB$_{E143A}$	MreB$_{E143A}$ Δ*rodZ*	<0.001
RodZ$_{1-337}$	RodZ$_{59-337}$	<0.001
RodZ$_{1-337}$	RodZ$_{70-337}$	<0.001
RodZ$_{1-337}$	RodZ$_{83-337}$	>0.05
RodZ$_{1-337}$	Δ*rodZ*	<0.001
RodZ$_{1-337}$	RodZ$_{1-155}$	<0.001
RodZ$_{1-337}$	RodZ$_{1-142}$	<0.001
RodZ$_{1-337}$	RodZ$_{1-111}$	>0.05
Δ*rodZ*	RodZ$_{59-337}$	<0.001
Δ*rodZ*	RodZ$_{70-337}$	<0.001
Δ*rodZ*	RodZ$_{83-337}$	<0.001
Δ*rodZ*	RodZ$_{1-155}$	>0.05
Δ*rodZ*	RodZ$_{1-142}$	<0.001
Δ*rodZ*	RodZ$_{1-111}$	<0.001

Supplementary References

1 Amann, E., Ochs, B. & Abel, K.-J. Tightly regulated *tac* promoter vectors useful for the expression of unfused and fused proteins in *Escherichia coli*. *Gene* **69**, 301-315 (1988).

2 Morgenstein, R. M. et al. RodZ links MreB to cell wall synthesis to mediate MreB rotation and robust morphogenesis. *Proc. Natl. Acad. Sci. USA* **112**, 12510-12515 (2015).

3 Ouzounov, N. et al. MreB orientation correlates with cell diameter in *Escherichia coli*. *Biophys. J.* **111**, 1035-1043 (2016).
