# Supplementary material for: MreB polymers and curvature localization are enhanced by RodZ and predict E. coli's cylindrical uniformity
Source: Nat Commun. 2018 Jul 18;9:2797. doi: 10.1038/s41467-018-05186-5 (PMC6052060; doi:10.1038/s41467-018-05186-5)
Supplement: Supplementary file 3 — Description of Additional Supplementary Files [file 41467_2018_5186_MOESM3_ESM.pdf]

Description of Additional Supplementary Files

File Name: Supplementary Data 1
Description: Excel file with 30 columns and 20 rows, with headers. Each row corresponds to one particular strain and the columns have the data values used for the LASSO analysis. See Supplementary Table 2 for expanded descriptions of the values.
